# A neurophysiological signature of dynamic emotion recognition associated with social communication skills and cortical gamma-aminobutyric acid levels in children

**DOI:** 10.3389/fnins.2023.1295608

**Published:** 2023-12-18

**Authors:** Daniela Sousa, Ana Ferreira, Diana Rodrigues, Helena Catarina Pereira, Joana Amaral, Joana Crisostomo, Marco Simoes, Mário Ribeiro, Marta Teixeira, Miguel Castelo-Branco

**Affiliations:** ^1^Coimbra Institute for Biomedical Imaging and Translational Research CIBIT, University of Coimbra, Coimbra, Portugal; ^2^Institute for Nuclear Sciences Applied to Health ICNAS, University of Coimbra, Coimbra, Portugal; ^3^Faculty of Medicine, University of Coimbra, Coimbra, Portugal; ^4^Centre for Informatics and Systems, University of Coimbra, Coimbra, Portugal; ^5^Department of Psychology, University of Maastricht, Maastricht, Netherlands

**Keywords:** social cognition, social communication, dynamic emotional expressions, event-related potentials, GABA, typically developing children

## Abstract

**Introduction:**

Emotion recognition is a core feature of social perception. In particular, perception of dynamic facial emotional expressions is a major feature of the third visual pathway. However, the classical N170 visual evoked signal does not provide a pure correlate of such processing. Indeed, independent component analysis has demonstrated that the N170 component is already active at the time of the P100, and is therefore distorted by early components. Here we implemented, a dynamic face emotional paradigm to isolate a more pure face expression selective N170. We searched for a neural correlate of perception of dynamic facial emotional expressions, by starting with a face baseline from which a facial expression evolved. This allowed for a specific facial expression contrast signal which we aimed to relate with social communication abilities and cortical gamma-aminobutyric acid (GABA) levels.

**Methods:**

We recorded event-related potentials (ERPs) and Magnetic Resonance (MRS) measures in 35 typically developing (TD) children, (10–16 years) sex-matched, during emotion recognition of an avatar morphing/unmorphing from neutral to happy/sad expressions. This task allowed for the elimination of the contribution low-level visual components, in particular the P100, by morphing baseline isoluminant neutral faces into specific expressions, isolating dynamic emotion recognition. Therefore, it was possible to isolate a dynamic face sensitive N170 devoid of interactions with earlier components.

**Results:**

We found delayed N170 and P300, with a hysteresis type of dependence on stimulus trajectory (morphing/unmorphing), with hemispheric lateralization. The delayed N170 is generated by an extrastriate source, which can be related to the third visual pathway specialized in biological motion processing. GABA levels in visual cortex were related with N170 amplitude and latency and predictive of worse social communication performance (SCQ scores). N170 latencies reflected delayed processing speed of emotional expressions and related to worse social communication scores.

**Discussion:**

In sum, we found a specific N170 electrophysiological signature of dynamic face processing related to social communication abilities and cortical GABA levels. These findings have potential clinical significance supporting the hypothesis of a spectrum of social communication abilities and the identification of a specific face-expression sensitive N170 which can potentially be used in the development of diagnostic and intervention tools.

## 1 Introduction

Humans gather substantial social information from faces (Adolphs, 1999). Facial emotional analysis includes conjugation of subtle facial gestures (Puce et al., 1998) which are processed in the third visual pathway (Pitcher and Ungerleider, 2021). Moreover, it is through fast perceptual processing of these changeable face stimuli in posterior STS (pSTS) posterior Superior Temporal Sulcus and later top-down processes in temporoparietal regions of the brain, involving attention and memory, that social communication is possible (Niznikiewicz, 2013; Liu et al., 2018). Prior EEG and imaging studies (e.g., Graewe et al., 2012; Bernardino et al., 2013; Castelhano et al., 2015) and in particular simultaneous EEG-fMRI studies provided evidence for a separable neural network underlying high-level facial expression recognition with a core hub at the pSTS (Simões et al., 2018, 2020; Direito et al., 2019; Abreu et al., 2020).

Most empirical studies about emotion recognition were driven by static faces, that do not require the visual system to integrate naturalistic movement paths (Tcherkassof et al., 2007; Furl et al., 2010; Harms et al., 2010; Krumhuber et al., 2013; Recio et al., 2014; Monteiro et al., 2017; Simões et al., 2018; Quadrelli et al., 2019). However, the analysis of facial emotions often requires deciphering the dynamic conjugation of facial gestures, from eye gaze to mouth movements, which is an important part of social communication (Puce et al., 1998; Key et al., 2022). Thereby, the use of tasks resembling more naturalistic demands will allow to generate more ecologically meaningful findings that can improve the generalization to the social communication context (Tcherkassof et al., 2007; Krumhuber et al., 2013; Sollfrank et al., 2021). A review about the effects of using dynamic aspects of facial expressions found that these dynamic features increase the consistency in identifying emotions, the intensity and arousal drove by emotional judgments, and facilitate the differentiation of genuine and fake expressions (Krumhuber et al., 2013).

One non-invasive way to study how the brain processes facial expressions at high temporal resolution is through event-related potentials (ERPs) recordings (Dzhelyova et al., 2017; Monteiro et al., 2017; Liu et al., 2018; Schindler and Bublatzky, 2020; Aydin et al., 2023). The N170 is a face sensitive ERP and has been related to high-level visual processes (Kuefner et al., 2010; Farashi et al., 2023). It is an occipitotemporal negative component, with a peak latency around 170 ms after the stimulus onset, which typically shows right-hemisphere lateralization (Kuefner et al., 2010). Moreover, neuroimaging data supports the notion that the right hemisphere dominates emotion recognition processing (Adolphs et al., 2000; Le Grand et al., 2003). Accordingly, the activation of the right superior temporal sulcus (STS) is specific to the attention given to facial emotions (Narumoto et al., 2001), which adds to the known dominance of the right fusiform face area (FFA; Apicella, 2013; Mason et al., 2022; Farashi et al., 2023). However, there is recent evidence for a third visual pathway specialized for the dynamic aspects of social perception, which includes distinct functional regions segregated from the ventral pathway (Pitcher and Ungerleider, 2021).

It is recognized that interpreting these ERPs pose challenges in the interpretation analysis due to the superposition of field potentials (Desjardins and Segalowitz, 2013). Accordingly, the early occipital component P100, which is sensitive to low-level properties of the stimulus, may modulate and affect the N170 amplitude and topography due to the temporal order of visual evoked potentials (Kuefner et al., 2010; Farashi et al., 2023). Thus, it is important to consider the influence of the P100 component on the N170, when designing experimental paradigms. In fact, the development of experimental designs that can capture the dynamic nature of social processes, such as face changeable features that occur during social communication interactions, is of utmost importance. This not only enhances ecological validity but also allows a more naturalistic approach to social cognition. The ability to isolate the N170 face effect source from the P100 effects is particularly relevant for studying face-related processing.

The spatial and temporal overlap of multiple electrocortical generators projecting to posterior scalp regions within 200 ms of the stimulus onset was previously investigated in the context of the face-effect timing issue through independent component analysis (ICA; Desjardins and Segalowitz, 2013). ICA is indeed the best method for blindly separating a set of mixed signals, and it allowed to unmix the field projections that constitute the P100 and N170 scalp ERP complex which constituent processes were shown indeed to overlap. The best way to avoid this mixing is to prevent the existence of one of the components.

Moreover, face processing paradigms are important to understand impaired social and emotional cognition in disorders, such as autism (Horder et al., 2013; Carvalho Pereira et al., 2018), schizophrenia, and major depressive disorder (Schür et al., 2016). These deficits have been ascribed to an imbalance between cortical glutamate excitation and gamma-aminobutyric acid (GABA) inhibition (Horder et al., 2013; Carvalho Pereira et al., 2018). GABA is the major inhibitory neurotransmitter (Edden et al., 2014; Carvalho Pereira et al., 2018) and is associated with neural synchrony, also with relevant physiological and higher-order processes (e.g., attention; Horder et al., 2013; Edden et al., 2014; Rae, 2014; Ende, 2015). Hence, the combination of ERP recordings, Magnetic Resonance Spectroscopy (MRS) and neuropsychological assessments may potentially illuminate the understanding of electrophysiological mechanisms involved in dynamic emotion recognition processes.

In the present study, we investigated the hypothesis that a neural signal specifically related to facial expression processing could be separated from low-level components, and that this specific signal could be related to socioemotional abilities and GABA neurotransmission. This work is motivated by prior findings that show a relationship between GABA and socioemotional cognition, particularly in the context of a clinical model of social impairments, such as autism (Carvalho Pereira et al., 2018). We tried to find a neural correlate of social and emotional cognition that we could relate to GABA and behavior. Hence, we predict that obtaining a specific neurophysiological signal evoked by a dynamic face emotional paradigm, would allow us to establish correlations with social communication abilities measures. Besides, we expect to find a correlation between the social communication abilities and GABA, supported by previous studies (e.g., Carvalho Pereira et al., 2018). Therefore, we predict that GABA linked to excitatory-inhibitory balance and face processing would be correlated not only with behavioral measures of socioemotional abilities, but also with a face processing specific neural signal. Previous studies have successfully provided evidence of neurodevelopmental disorders relation with GABA, neurophysiology, and behavior (e.g., Ribeiro et al., 2015). In this way, our goal was to provide a direct link of face processing of dynamic expressions with socioemotional cognition and neurochemistry in typically developing (TD) children. Thus, by presenting evidence of these correlates in TD children, we can support the hypothesis of a spectrum within neurotypical development which could offer valuable insights for future directions in autism research. By using a multimodal approach, we sought to investigate specific facial expression selective ERPs elicited by a morphing avatar in TD children. Our goal was to remove the contribution and/or interaction of early (low-level) P100 with N170 component to derive a specific facial expression neural signal.

It is increasingly being recognized that multimodal approaches might help to deepen our understanding of emotion recognition processing. Here we used such a strategy, while considering that the use of static face stimulus paradigms fails to mimic the processing required by discrimination of subtle facial expressions in everyday interactions. Therefore, the study of facial emotion processing requires the use of ecologically valid stimuli and the consideration of timing effects, which will allow the interpretability of ERPs effects at distinct latencies (Desjardins and Segalowitz, 2013). This is because the classical face evoked N170 may have contributions from both low- and high-level processing components, and that to isolate a specific emotion recognition component a novel paradigm is needed. Such a paradigm would be particularly relevant to separate the N170 face-expression sensitive component from the early P100 effects. This is because early visual effects intermingle with higher-level processes, which has been clearly demonstrated by ICA (Desjardins and Segalowitz, 2013). It is therefore of utmost importance to disentangle the P100 effect stemming from low level features from the N170 component. Therefore, in order to isolate that neural signal, we used a dynamic facial expression recognition task with a virtual avatar morphing from an isoluminant neutral expression baseline to full expression and subsequently unmorphing to neutral, as adapted from a previous design of Simões et al. (2018). By including a static face in the baseline and keeping stimulus local luminance and contrast levels constant from baseline to dynamic stimulus onset, we tried to remove early components. The absence of a P100 component would show that this strategy might be successful. An important point is that removing one component would improve the estimation of subsequent component. This is due to the potential interaction of overlapping components, which may affect amplitude and latency estimation. Our strategy to experimentally remove one component would solve this potential overlap problem, and yield a specific neural signal to be related with GABA signals and socioemotional cognition. We investigated the temporal effect of morphing versus control unmorphing sequences (which are reversed versions of each other in time). We examined morphing/unmorphing sequences to identify the N170 cortical generators.

This approach allowed to investigate the relation between social communication abilities, neural inhibition as probed by GABA levels and neurophysiological measures of facial expression processing.

## 2 Materials and methods

### 2.1 Protocol approvals and participants’ consents

Written informed consent was obtained from parents and children. The study was approved by the ethics committee from Faculty of Medicine, University of Coimbra (UC) and was conducted under the declaration of Helsinki.

### 2.2 Participants

Thirty-five TD children [18 females and 17 males; mean (*M*) age = 13.06 and standard deviation (*SD*) = 1.91] were recruited from our local volunteers’ database, schools, and the community at Coimbra, Portugal. All caregivers underwent a clinical interview conducted by a clinical psychologist and completed the social communication questionnaire (SCQ, Supplementary Information, see Instruments) to evaluate their children’s social communication abilities (Rutter et al., 2003). Participants had no reported history of either neurodevelopmental or neurological disorders. The inclusion criteria were children or adolescents with a typical development with ages between 10 and 16 years old. The exclusion criteria included obtaining a score higher than 15 in the SCQ questionnaire and previous or present story of neurological diseases/brain surgery and/or neuropsychiatric disorder. Table 1 shows the group characterization.

**Table 1:** Group characterization.

Measure	Mean (*M*)	Standard deviation (*SD*)
FSIQ	120.80	14.61
VIQ	123.77	16.14
PIQ	112.17	11.84
Social communication questionnaire – total (SCQ – T)	3.74	3.33
SCQ – communication (SCQ-C)	1.37	1.86
SCQ – Reciprocal social interaction (SCQ – RSI)	0.80	1.47
SCQ – Repetitive/stereotyped behavior (SCQ – R/SB)	0.23	0.55

The participants intelligence quotient (IQ) was assessed by the Wechsler Intelligence Scale for Children – 3rd Edition (WISC-III): mean (M) and standard deviation (SD) for the full-scale intelligence quotient (IQ) and SCQ scores. IQ (FSIQ), verbal IQ (VIQ) and performance IQ (PIQ) are presented. As well as the social communication questionnaire (SCQ) scores for the Total score (SCQ-T) and subscales: Communication (SCQ-C), Reciprocal social interaction (SCQ-RSI), and Repetitive/Stereotyped behavior (R/SB).

### 2.3 Facial emotion recognition task

**Figure 1:**
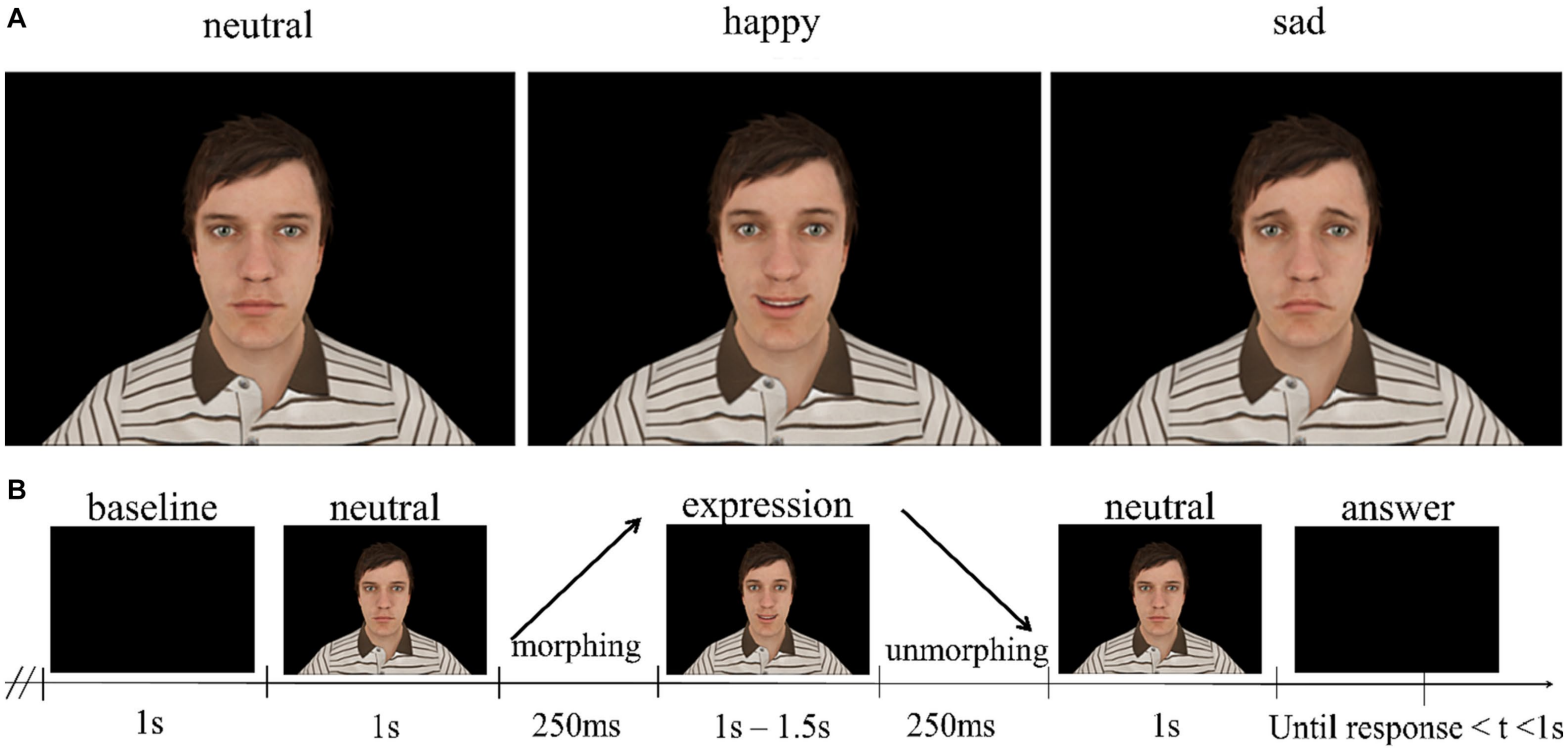
**(A)** Facial expressions used as stimuli in the experiment. **(B)** Structure of the trials. Happy and sad facial expressions took 1.5 s, separated by facial expression morphing, static facial expression presentation and facial expression unmorphing. The visual stimuli and paradigm were developed in WorldViz Vizard 5 VR Toolkit (development edition) using the male002 virtual avatar from the Complete Characters HD pack and its FE poses.

The visual task was developed using the *Matlab^®^ (Mathworks, version R2017a)* with the *male002* virtual avatar from the *Complete Characters HD pack* and its facial expressions poses (Figure 1A), for details see Simões et al. (2018).

Each trial is composed of the presentation of a baseline period with a virtual avatar already displaying a neutral expression (1 s – 1.5 s), followed by a morphing period of 250 ms where the avatar gradually transitions from neutral expression to the target one (providing a direct contrast between neutral and emotion expressing faces). Then it is followed by a period where the virtual avatar is maintaining the target facial expression (happy or sad) in its full extent (1 s), and a final period of 250 ms where the avatar morphs back (unmorphs) to the neutral expression, which is presented with a non-fixed random duration (between 1 s to 1.5 s). To ensure unpredictability, the task incorporates the randomization of emotional expressions, and the distinct randomization within each run. Additionally, it was specified that each condition (sad or happy) could only appear in a maximum of three consecutive trials. The participants were asked to fixate the face of the avatar in the middle of the eyes, observe the expressions and decide whether the avatar displayed a happy or sad expression by pressing one of two buttons after the avatar morphs back to the neutral expression for 1 s maximum (Figure 1B). This experiment consisted of 3 runs of about 4 min (50 trials per run – 25 happy and 25 sad), with short breaks in between to ensure focus and reduce fatigue throughout the task. The experiment total duration was about 40 min.

Our previous studies (e.g., Graewe et al., 2012; Bernardino et al., 2013; Castelhano et al., 2015) and in particular our EEG-fMRI studies provided evidence for a separable neural network underlying high-level facial expression recognition (Simões et al., 2018, 2020; Direito et al., 2019; Abreu et al., 2020). In the current experiment, we added an unmorphing condition which has the same quantity of motion as the morphing one. We predict that the morphing condition should lead to higher activation due to the evolving facial expression. If this holds true and considering that the amount of low-level motion remains the same, it would provide additional evidence for a high-level facial expression signal. Therefore, we aim to demonstrate this by incorporating a specific face expression contrast in our experimental design. This is only possible to achieve if in the baseline a face is already present, and a facial expression then evolves. Since the same low-level features are present in the baseline, no P100 will be elicited upon appearance of a facial expression. Specifically, we anticipated that the neural response to the emotionally morphed expression would be elicited in the absence of an interfering P100. Hence, this will allow to study the facial emotional processing without the visual contamination provided by early visual signals.

### 2.4 Electroencephalography acquisition and processing

The experiment was conducted in a 22-inch LCD monitor (frame rate of 60 Hz, 1680×1050 pixel resolution) and the paradigm was delivered using *Matlab^®^ (Mathworks, version R2017a)*. The participants sat about 60 cm away from the screen.

EEG data were recorded using the actiCAP with 64 Ag/AgCl active electrodes, according to the international 10–10 standard system directly connected to the Brain Products actiCHamp amplifier and sampled at 1000 Hz. The ground electrode was positioned at AFz and reference electrode was at the FCz position. The electrodes impedance was kept under 15 KΩ. EEG signals were recorded using the BrainVision Recorder software (*Brain Products, version 1.20.0801*).

We used *Matlab^®^* (*Mathworks, version R2019b*) and the EEGLAB toolbox v2019_0 (Delorme and Makeig, 2004) for EEG signal pre-processing and analysis. EEG data were filtered with a finite response bandpass filter with lower and higher cut-off frequencies set to 0.1 Hz and 30 Hz, respectively. Bad channels were then removed by visual inspection (2 to 3 channels, on average) below the standard 5% limit (Picton et al., 2000) and then interpolated. Afterwards, data were re-referenced to the common average reference. Epochs were created time-locked to the onset of the morphing of the target facial expression, beginning 2 s before and lasting up to 3.5 s after. Bad epochs were removed based on the EEGLAB semi-automatic procedures for extreme values and improbable signal segments. On average 94.91% of the trials remained for further analysis, from which 94.67% were happy and 93.76% were sad facial expressions trials. Independent Component Analysis (ICA) was then run on data using EEGLAB implementation of *infomax* algorithm (Bell and Sejnowski, 1995) to extract noisy components (e.g., blinks). Noisy components were identified using the EEGLAB plug-in ICLabel, removed and the weights of the remaining components were projected back to the data (Makeig et al., 2004). Further analysis of EEG data was conducted over these pre-processed signals.

ERPs were computed across lateral posterior-temporal and parieto-occipital sites (P3, P4; PO7, PO8) based on previous studies (e.g., De Jong et al., 2008; Kuefner et al., 2010), by averaging trials grouped by expression and static/neutral, morphing and unmorphing conditions (simple baseline correction of 200 ms before the stimulus onset). According to Simões et al. (2018), we defined a window of 150–350 ms and 300–700 ms following face stimulus onset (i.e., neutral, happy, or sad) or expression trajectory (i.e., neutral/static, morph or unmorph). The peak values were automatically detected as local minima for negative waves, or local maxima for positive waves. For each participant, grand average peak amplitudes and latencies were calculated for each component.

### 2.5 Electroencephalography source estimation

Source analysis was performed using the Curry 7.0.8 software (Neuroscan, United States) by importing the pre-processed grand averages by type of biological motion (morphing and unmorphing). ERP data were coregistered with an anatomical standardized Boundary Element Method model (BEM), implemented according to Curry software when working without individual image data, and the standard electrode’s position. Then, a Current Density Reconstructions (CDR) was conducted based on sLoreta (standardized low-resolution brain electromagnetic tomography; Pascual-Marqui, 2002). The sLoreta yields images of standardized current source density of a total of 9,627 sources. The results were obtained using the Montreal Neurological Institute/Statistical Parametric Mapping (99; MNI/SPM99) coordinates with 80% of the power.

### 2.6 Acquisition of magnetic resonance imaging data

**Figure 2:**
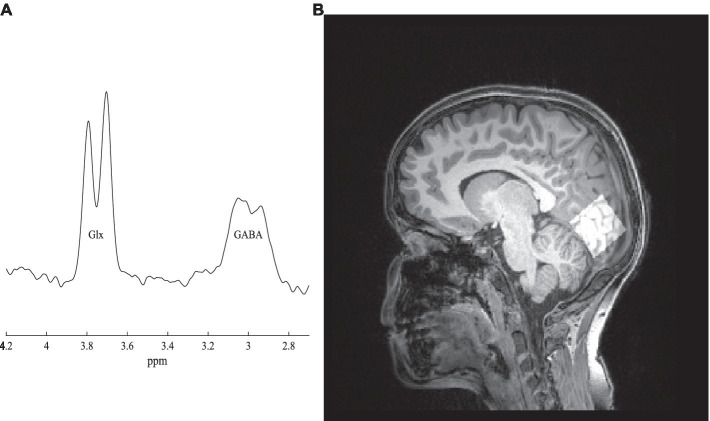
**(A)** GABA spectrum. **(B)** The voxel localized in the occipital cortex (right side).

MRI experiments were conducted with a 3 T Siemens Magnetom Prisma MRI Scanner (Siemens, Erlangen, Germany) at Institute for Nuclear Sciences Applied to Health (ICNAS), UC. For each participant, a high-resolution T1-weighted three-dimensional Magnetization Prepared Rapid Acquisition Gradient Echo (MPRAGE) sequence [repetition time (TR) 2,530 ms, echo time (TE) 3.5 ms, inversion time (TI) 1,100 ms, flip angle (FA) 7°, field of view (FOV) 256 × 256 mm^2^, yielding 192 slices with 1 × 1 × 1 mm^3^ voxel size and 1 mm of thickness] was firstly performed for structural assessment and localized 1H-MRS voxel placement. 1H-MRS data were collected on a volume of interest placed on the occipital cortex (voxel size: 30 mm x 30 mm x 30 mm), positioned accordingly with sagittal, coronal, and axial planes to minimize partial volumes effects (Figure 2B). GABA and Glx (Glutamate+Glutamine) measurements were carried out using the Hadamard Encoding and Reconstruction of Mega-Edited Spectroscopy (HERMES) approach, as implemented by Chan et al. (2016) with parameters defined as follows: TR = 2000 ms, TE = 80 ms, number of averages = 320, flip angle = 90^o^, bandwidth = 2000 Hz. An unsuppressed water signal (TR = 2000 ms, TE = 80 ms, number of averages = 32, flip angle = 90^o^, bandwidth = 2000 Hz) was acquired immediately after acquiring the water-suppressed spectrum. The total acquisition time was approximately 20 min. All participants watched videos during the scanning protocol to help them remain still during the acquisition period.

#### 2.6.1 1H-MRS data processing and quality check

Data were saved as TWIX files and processed using *Matlab® (MathWorks, version R2019a)* with the default pipeline implemented in Gannet toolbox v3.0 (Edden et al., 2014). Spectra were firstly inspected for movement artifacts and corrected for frequency drift. A difference spectrum was generated per participant and peak integration was used to quantify GABA (3.0 ppm) and Glx (3.75 ppm; Figure 2A). Here, the signal corresponding to GABA is labeled GABA+ to indicate the potential contribution of macromolecules and homocarnosine at 3.02 ppm (Rothman et al., 1997). Integrals of GABA+, Glx and total creatine (tCr) peaks were automatically calculated using a Gaussian (GABAþ), Gaussian doublet (Glx) and Lorentzian (tCr) models to best fit the peaks, as implemented in the toolbox. Relative proportions of gray matter, white matter, and cerebrospinal fluid, in the voxel, were obtained by performing tissue segmentation of T1-weighted images using the same software and SPM12 toolbox.^1^ Additionally, these proportions were used to adjust metabolite levels to correct for different voxel compositions. Moreover, segmentation reduces inter-subject variability attributable to differences in signal-to-noise ratio, regional susceptibility variations and cerebrospinal fluid fraction within the voxel (Bogner et al., 2010). Finally, absolute quantification of GABA and Glx concentrations were taken relative to water peak, therefore expressed in institutional units (i.u.).

### 2.7 Statistical analysis

All statistical analyses were performed in IBM Statistical Package for the Social Sciences (SPSS), Version 25. We calculated the overall behavioral data accuracy (Supplementary Information, see Behavioral Data). The normality assumption was verified using the Shapiro–Wilk test. Most ERPs parameters were normally distributed, hence a two-way repeated Analyses of Variance (ANOVA) were performed. We investigated the ERPs (amplitude and latency) elicited by a morphing avatar of facial expressions in TD children using the facial expressions (neutral, happy, and sad) and hemisphere (P3 or P4 sites) as within-subject factors. Additionally, we explored the temporal trajectory (morphing) effect on ERPs (amplitude and latency), where the expression trajectory (morph and unmorph trajectories, as well as neutral/static) and hemispheres (PO7 or PO8 sites) were introduced as within-subjects factors. Greenhouse–Geisser adjustments were used for sphericity assumption violations. Bonferroni adjustment for pairwise comparisons was applied and partial *η2* was calculated to estimate effect sizes.

Finally, Spearman’s rho correlations were computed to explore the relationships between ERPs and GABA+ with the participants’ social communication skills measured by SCQ. Likewise, the False Discovery Rate (FDR) was applied using the Benjamini-Hochberg procedure for multiple comparisons correction, with a critical value of 0.25 (Benjamini and Hochberg, 1995). An alpha level of 5% was used as the statistical significance threshold.

Regarding the exclusion of data due to poor signal-to-noise ratio, 7 participants were excluded from the EEG analysis, 5 participants from the MRS analysis and another 5 participants did not perform the MRI for other reasons (e.g., could not tolerate the MRI). Thus, the final sample sizes were 28 and 25 for the EEG and MRS analyses, respectively.

## 3 Results

### 3.1 An N170 face sensitive ERP specific to facial expressions without P100 contamination

**Figure 3:**
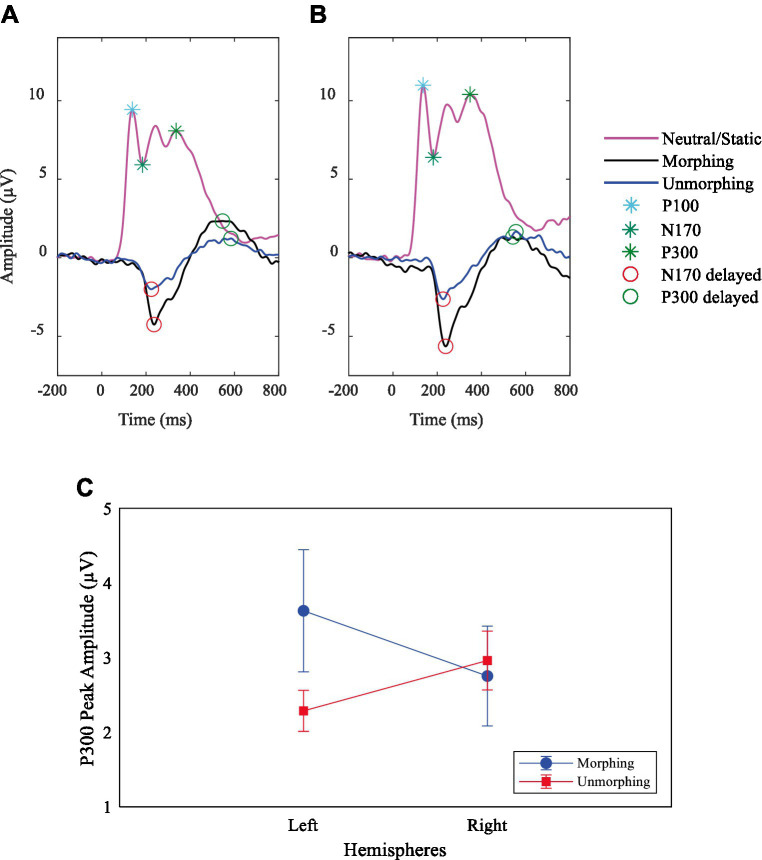
Demonstration of removal of influence from P100 on specific facial expression ERPs. Grand average ERPs plots for P100 and N170 components on neutral expression and delayed N170 and P300 at site **(A)** PO7 (left hemisphere) and **(B)** PO8 (right hemisphere) for biological motion of facial expressions. **(C)** P300 peak amplitude interaction effect between the stimulus type, left (Channel PO7) and right (Channel PO8) hemispheres.

This paradigm enabled a specific contrast between dynamic expressions and an isoluminant neutral expression baseline from which they were morphed, leading to a dynamically delayed N170, without P100 (driven by low-level features) contamination. Figures 3A,B shows a “pure” N170 (devoid of interference of low-level features) evoked by morphing and unmorphing facial expression trajectories. Additionally, Supplementary Figure S1 further demonstrates this “pure” N170 evoked by both happy and sad emotional expressions, derived from the contrast between morphing a facial expression and neutral, by removing the masking effect of P100. A main effect of type of expression on N170 amplitude was verified, *F* (1.31, 35.35) = 13.74, *p* < 0.001, *η^2^_p_ = 0*.34, with larger amplitudes for happy (*M =* −3.36, standard error *SE* = 0.29) and sad (*M* = −2.67, *SE* = 0.31) than the control neutral expression (*M =* −0.66, *SE* = 0.46; see also Supplementary Figures S1–S5).

Regarding the ERPs amplitude and latency for happy and sad expressions, we found only a main effect of facial expressions on P300 latency, *F* (2, 54) = 100.91, *p* < 0.001, *η^2^_p_ = 0*.79, showing longer latencies for the sad expression (*M* = 575.07, *SE* = 13.75) compared to both the happy (*M* = 503.50, *SE* = 15.81) and the neutral expressions (*M* = 353.75, *SE* = 8.99).

### 3.2 Morphing expression produces larger neural responses than unmorphing: the effect of inverting expression trajectories

Grand average waveforms at parieto-occipital electrodes yielded a main effect of expression trajectory (neutral, morphing and unmorphing) for the N170 amplitude (see Supplementary Figures S1, S3A,B). The amplitude was higher for morphing (*M* = −5.86, *SE* = 0.49, *F* (1, 27) = 131.94, *p* < 0.001, *η^2^p* = 0.83), as opposed to the unmorphing (*M* = −3.05, *SE* = 0.20), *F* (1, 27) = 67.54, *p* < 0.001, *η^2^p* = 0.71, when compared in magnitude with the neutral ones (*M* = 3.32, *SE* = 0.74). Most importantly, comparisons for morphing and unmorphing, *F* (1, 27) = 45.31, *p* < 0.001, *η^2^p* = 0.63, disclosed that amplitude was significantly higher for morphing (*M* = −5.86, *SE* = 0.49) than unmorphing (*M* = −3.05, *SE* = 0.20).

Regarding the N170 latency, a main effect of expression trajectory was also found, *F* (1, 27) = 8.09, *p* = 0.008, *η^2^p* = 0.23, with longer latencies for morphing (*M* = 248.75, *SE* = 7.73) than neutral (*M* = 217.82, *SE* = 9.32), corroborating the delayed N170 characteristic of facial expressions. Also, comparisons between neutral and unmorphing, *F* (1, 27) = 10.78, *p* = 0.003, *η^2^p* = 0.29, confirmed that latency was larger for unmorphing (*M* = 251.79, *SE* = 6.81) than neutral (*M* = 217.82, *SE* = 9.32).

Concerning the P300 amplitude, we found that neutral and unmorphing expressions showed a right hemispheric bias, while morphing showed a left bias (Supplementary Figures S6–S7; Figure 3C). The comparison between morphing and unmorphing showed an interaction effect with hemispheres, *F* (1, 27) = 12.10, *p* = 0.002, *η^2^p* = 0.31; Figure 3C.

Regarding the P300 latency, we observed a main effect of expression trajectory, *F* (1, 27) = 253.99, *p* < 0.001, *η^2^p* = 0.90, disclosing longer latencies for morphing (*M* = 534.86, *SE* = 11.69) than neutral (*M* = 352.00, *SE* = 7.30), as expected from the dynamic emotional delay effect. Additionally, the comparisons between neutral and unmorphing, *F* (1, 27) = 224.69, *p* < 0.001, *η^2^p* = 0.89, confirmed that latency was larger for unmorphing (*M* = 557.14, *SE* = 15.15) than neutral (*M* = 352.00, *SE* = 7.30). Moreover, a main effect of hemispheres was observed, *F* (1, 27) = 4.95, *p* = 0.035, *η^2^p* = 0.16, with higher latencies for the right (*M* = 468.04, *SE* = 9.87) than the left side (*M* = 441.11, *SE* = 12.85).

#### 3.2.1 Source analysis

**Figure 4:**
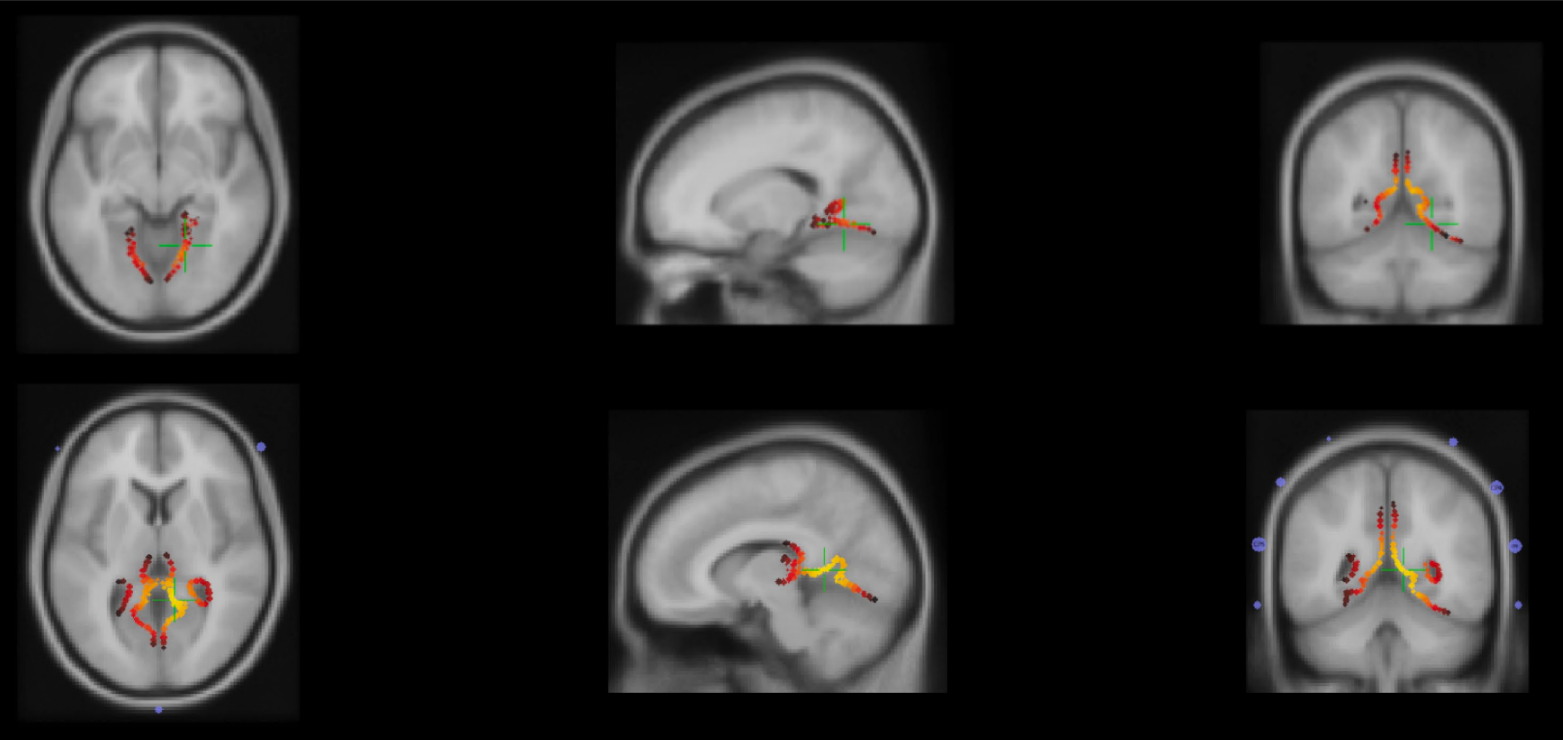
Top: Axial, sagittal and coronal planes (from left to right) of secondary visual cortex, inferior occipital gyrus activation due to N170 response to the neutral/static condition identified by the green plus signal. Bottom: Axial, sagittal and coronal planes (from left to right) of secondary visual cortex, middle occipital gyrus activation due to morph and unmorph conditions identified by the green plus signal.

The CDR results (sLoreta) for neutral N170 showed activation in the right extrastriate visual cortex at the inferior occipital gyrus with the coordinates MNI/SPM99 (17.8, −56.9, −8.1) mm and variance explained of 97.91% (Figure 4).

Additionally, the delayed N170 signal obtained during both morph and unmorph conditions suggests the involvement of the same brain source, due to the activation of the right extrastriate visual cortex at the middle occipital gyrus, with the coordinates MNI/SPM99 (10.2, −48.4, 6.0) mm and with variance explained of 95.59% for the morph and for the 91.96% unmorph (Figure 4).

### 3.3 Correlations between GABA levels, neurophysiological measures and social communication skills

We found important neurobehavioral correlations between GABA levels, neurophysiological responses and social behavior. Concerning Spearman’s rho correlations between GABA and ERPs for facial expressions and biological motion, we found a significant pattern of associations that remained significant after FDR correction. GABA concentrations were positively correlated with the amplitude of N170 (μV) for unmorphing sad and happy facial expressions (for the left hemisphere), *r_s_* = 0.45, *p* < 0.05, *n* = 25, and negatively correlated with P300 latency (ms) to sad morphing expressions (left hemisphere), *r_s_* = −0.55, *p* < 0.01, *n* = 25. These results seem to corroborate the role of excitatory-inhibitory balance in face processing.

**Figure 5:**
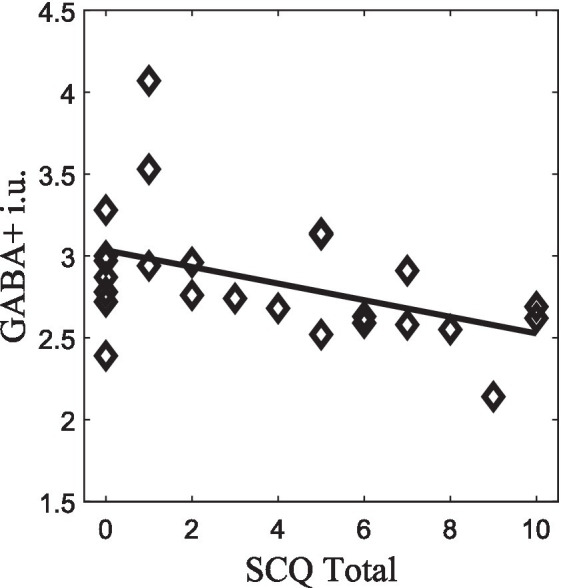
GABA+ correlation with SCQ-Total score (*p* < 0.05), which survived to FDR correction.

GABA+ concentrations also showed significant correlations with behavioral features. Regarding the SCQ, we found that the SCQ-Total score was negatively correlated with GABA+ levels, *r_s_* = −0.49, *p* = 0.014, *n* = 25 (Figure 5). Additionally, correlations results between SCQ and ERPs for facial expressions are summarized in Supplementary Table 1, highlighting the notion of worse scores in SCQ impair speed of processing of sad and happy emotional expressions. Regarding associations between the direction of facial expression responses (morphing/neutral/unmorphing) and SCQ, they are shown in Supplementary Table 2. These results stress the complex interplay among electrophysiological responses, neurotransmitter’s role, and social communication behavior studied in disorders, such as autism.

## 4 Discussion

This work was motivated by our prior observation that GABA is related to socioemotional cognition in a clinical model of social impairments such as autism (Carvalho Pereira et al., 2018). Here, we attempted to identify a neural correlate of social and emotional cognition that we could relate to GABA and behavior. The inference of emotional signals is critical for social communication (Apicella, 2013; Meaux et al., 2014; Dzhelyova et al., 2017; Li et al., 2019; Schindler and Bublatzky, 2020; Key et al., 2022). In this study, we used a multimodal approach on a large sample of sex-matched TD children to establish neurobehavioral and neurochemical relationships with neurophysiological signatures of dynamic facial expression recognition, where early visual components could be discounted for, yielding a specific facial expression signal. We found significant associations between social communication skills, neurophysiology, and neurochemistry in emotional processing.

Desjardins and Segalowitz (2013) have previously identified multiple electrophysiological processes that accounted for face-related ERP effects at the scalp during the period of the P100 and N170 complex, which are all active over the period of both the P100 and N170 time periods. It was therefore important to find methods for removing the P100 component, overcoming the necessity of unmixing the scalp signals to enable correct interpretation of scalp voltage differences.

We found that the P100 response observed for the neutral expression could be removed from the emotional expressions evoked ERP, thereby suppressing the interference of early signals from the primary visual cortex, yielding a purer emotion recognition signal. This result may be explained by the use of the isoluminant neutral expression as a baseline to guarantee a facial emotion-specific contrast (Simões et al., 2018). Additionally, it is known that features of the stimulus (e.g., brightness, luminance, contrast) can affect early ERPs (Mueller et al., 2013), which in this case was avoided by the presentation of physically matched isoluminant baseline face stimuli. Also, an important control in this study is the reversed temporal direction of morphing by creating an unmorphing condition. If the signal would simply reflect low-level motion it should remain constant. By using the unmorphing control condition, which has a quantity of motion that is equal to the morph, we showed that this is not the case. Indeed, the neural responses are smaller when the emotional expression is reduced irrespective of the fact that the amount of low-level motion remains the same. Moreover, previous EEG (Graewe et al., 2012; Bernardino et al., 2013; Castelhano et al., 2015) and EEG-fMRI studies (Direito et al., 2019; Abreu et al., 2020; Simões et al., 2020) have demonstrated separable neuronal networks underlying emotional expression processing, independent of early motion processing. In particular, the aforementioned EEG-fMRI studies allowed to reconstruct specific high-level sources of the facial expression network. The observation that extrastriate sources are right lateralized, is consistent with right hemispheric lateralization of social and emotion processing (Amaral et al., 2015).

In sum, the data from previous studies, along with the analysis shown in the current article, indicates that the observed effects cannot simply be due to motion because the quantity of movement is identical in both conditions. Only the amplitude change of facial expressions is different.

Importantly, we found a delayed N170 and P300 for emotional expressions. These results can be explained by the temporal properties of the stimuli. Dynamic facial emotion tasks require visual scrutiny of morphing features (Arsalidou et al., 2011), leading to processing speed constraints. Such analysis is likely to elicit top-down cognitive control as a result of the demand to integrate all the information induced by dynamic continuing information updates (Arsalidou et al., 2011). In this study the unmorphing of the facial expression, which is basically a temporal reversal of the stimulus from fully morphed at the beginning of stimulus presentation to fully unmorphed, had a similar waveform and latency to the morph, but lower amplitude. This lower amplitude, related to lower facial expression strength suggests that the delayed ERPs results from true processing of dynamic facial information. Besides, it has been reported that the occipital negativity around 150 ms to 350 ms reflects the transition to extrastriate and higher-level processing where task-relevant features are possibly selected (Schacht and Sommer, 2009). Besides, top-down attention processing at later stages seems to be required for affective relevant features, allowing a boosting emotional effect (Dzhelyova et al., 2017; Liu et al., 2018; Schindler and Bublatzky, 2020). Therefore, it has been proposed that during the processing of dynamic facial expressions attentional amplification occurs (Recio et al., 2014; Quadrelli et al., 2019). Further, the use of a dynamic facial emotion recognition task increases the ecological validity and resembles everyday interaction where we need to read subtle changes in facial emotion in others. However, a limitation is that dynamic avatar expressions may reflect an artificial facial motion, which could lead to differences in temporal electrocortical responses compared to those evoked by real faces, particularly slowing the ERPs (Sarkheil et al., 2013; Sollfrank et al., 2021).

We also found evidence for P300 interaction effects of the type of facial expression direction, suggesting hysteresis (morphing vs. unmorphing) and hemispheres. These results showed a consistently larger electrocortical response on the right hemisphere (Arsalidou et al., 2011; Dzhelyova et al., 2017; Leleu et al., 2018; Quadrelli et al., 2019) for neutral and unmorphing stimuli aligning with studies suggesting the right lateralization of face processing in both adults and children (Puce et al., 2000; Olofsson et al., 2008; Ibáñez et al., 2011; Leleu et al., 2018; Quadrelli et al., 2019). Besides, we found longer P300 latencies for the sad expression, potentially explained by the fact that it might recruit larger attentional resources (Leleu et al., 2018). Moreover, Recio et al. (2014) found that during an active task involving dynamic facial expressions it was more difficult to distinguish between neutral and sadness expressions (i.e., identify neutral as sad). This difficulty may arise from larger morphological similarity between the two expressions. This is supported by the fact that we had an active task which required a behavioral response from the participant, which could lead to a state of higher-levels of selective attention to discriminate subtle changes in the avatar facial expressions from neutral to sad. Moreover, higher attentional deployment is associated with late and longer ERPs (200–1,000 ms; Olofsson et al., 2008; Krumhuber et al., 2013).

Functional MRI studies have shown three main N170 sources: the occipital face area – OFA, the fusiform face area – FFA, and the posterior superior temporal sulcus face area – pSTS-FA (Olofsson et al., 2008). Our data revealed the contribution of the right inferior and middle occipital gyrus for the N170 during morphing and unmorphing. Moreover, the neuronal responses are smaller when the emotional expression is reduced (unmorphing), irrespective of the fact that the amount of low-level motion remains the same. Interestingly, morphing induced brain activity showed right hemispheric bias which is consistent with the known lateralization for strong socioemotional cues (Amaral et al., 2015). These results are consistent with the hypothesis that ventral stream regions, in particular OFA and FFA are involved in social cognition processes, specifically by having a role in the detection of faces, decoding facial motions and interpreting them as social cues (Arsalidou et al., 2011; Dzhelyova et al., 2017). Nevertheless, there is recent evidence of a functional dissociation between the ventral pathway, which includes the FFA and OFA and a possible third visual pathway responsible for the dynamic aspects of social perception, such as biological motion (e.g., facial and body movement; Pitcher and Ungerleider, 2021). This pathway drives from the early visual cortex, via motion-selective areas, into the STS showing a pathway projecting along the STS specialized for dynamic face perception (Pitcher and Ungerleider, 2021). Moreover, it seems to be also anatomically distinct from the ventral pathway being associated with social cognitive processing (Pitcher and Ungerleider, 2021). However, source data should be interpreted with caution due to EEG’s relatively low spatial resolution.

Finally, our results suggest a link between social cognition abilities and the biological substrates of emotion recognition. This is expressed by the fact that worse social communication scores lead to reduced processing speed. Accordingly, higher scores in SCQ-Total, SCQ-Communication and SCQ-Reciprocal Social Interaction subscales lead to longer latencies in N170 on the right hemisphere, which is a result consistent with EEG study’s findings in autism (Kang et al., 2018). The paradigm employed here increases ecological validity and allows for a more naturalistic social cognition approach, which can further elucidate the role of the face sensitive N170 in autism. Studies have shown delayed N170 latencies in individuals with autism, reflecting less efficient face processing or incomplete developmental maturation (Kala et al., 2021; Aydin et al., 2023; Farashi et al., 2023). Moreover, the right hemisphere N170 latency to upright faces was accepted into FDA Center for Drug Evaluation and Research (CDER) Biomarker Qualification Program (Webb et al., 2023). Further studies with this paradigm should be done with larger samples to explore the consistency of these results, as well as the potential use of N170 as a biomarker of treatment in autistic population conjugated with a naturalistic social cognition approach.

Furthermore, we found a negative correlation between GABA+ and measures of social communication, which seems to uncover that low GABA+ levels in face visual processing regions are related to worse social communications abilities. This result is also corroborated by studies in autism, characterized at its core by social dysfunction which found that GABA+, GABA+/tNAA and GABA+/tCR values were also negatively correlated with ADI-R communication score (Carvalho Pereira et al., 2018), which match the communication SCQ. Nonetheless, SCQ had not been extensively researched in the general population (Moody et al., 2017). These findings could enlighten future works on important features of the clinical phenotype of autism, which has a core deficits in social communication and interaction (Carvalho Pereira et al., 2018) and TD individuals exposing the relevance of studying these characteristics as a continuum of social communication abilities across individuals. The correlation analyses should be interpreted with caution since given the sample size that led to the decision to render them exploratory.

In sum, we found that GABA is negatively correlated with ERP latency, which means that longer latencies are associated with lower GABA levels. Deficits in social cognition have been implicated to an imbalance between cortical glutamate excitation and GABA inhibition (e.g., Devitt et al., 2015) and our results seem to corroborate this relation. Therefore, we found that higher scores in SCQ (meaning more difficulties in social communication) are associated with lower GABA levels, as well as longer latencies. These findings suggest a less efficient face processing, pointing out ERPs latency as a potential biomarker for social communication abilities. In contrast, we found a positive relation between N170 amplitude and GABA for unmorphing expression trajectory, which means that higher N170 amplitudes are associated with higher GABA levels, consistently with the notion that lower GABA levels are more detrimental. The fact that GABA is more specifically related to responses to fading visual stimuli can be related with the aforementioned push-pull of inhibitory versus excitatory processes.

Additionally, we found for sad facial expression and neutral/static expression trajectories a negative correlation between SCQ scores and P300 amplitudes. This implies that higher amplitudes in brain activity reflecting high-level social attention processing results in less impairments in social communication abilities. Therefore, we identified a specific neurophysiological signature of emotional recognition of dynamic facial expressions in TD children which is associated with social cognition measures. To the best of our knowledge, this is the first face processing paradigm that could elicit a pure face sensitive N170 component without the P100 modulation. This is an important contribution due to the intrinsic interpretation challenges implied by the mixing of electrocortical field potentials at the scalp. These challenges are amplified in the early face-related ERP differences, given the spatiotemporal field projections during the P100 and N170 that are highly overlapping. This novel specific marker showed to be related with brain neurochemistry of inhibition and social cognition skills. Future studies should elucidate the potential of this paradigm in autism and other disorders, where deficits in social communication occur, featuring the use of multimodal approaches, such as the combination of functional MRI, ERPs, and the integration of other psychophysiological measures. Concerning the implications of the results, they are relevant to understand the neural basis of socioemotional cognition and can be applied in the context of autism research. Additionally, this neural signal can also be investigated in the future as a biomarker in autism research, using the machine learning tools proposed by Simões et al. (2018). This means that having paradigms that focus on a core deficit and evoking a specific neural response will be a potential asset for biomarker development that can be used in future interventions in autism research. Given the role of impaired face emotional recognition as a core feature in autism, our results have potential clinical significance. Prior studies have already supported the notion that classical N170 latencies could be an indicator of autism [e.g., Aydin et al., 2023]. Moreover, Mason et al. (2022) provided results which emphasize the classical N170 component as a promising stratification marker in clinical trials, since N170 latencies have been associated to social difficulties from childhood to adolescence. Our novel facial expression specific N170, which likely targets more directly the third visual pathway, may improve even further this scenario. This is in line with our results in TD children and adolescents supporting the existence of a spectrum of social communication abilities, which have a neurophysiological and neurochemical substrate. Therefore, our findings have clinical implications regarding the future development of early diagnosis and/or identification tools, such as EEG based facial expressions paradigms, and intervention with children/adolescents, targeting emotion recognition and social skills.

## Data availability statement

The raw data supporting the conclusions of this article will be made available by the authors, without undue reservation.

## Ethics statement

The studies involving humans were approved by Comissão de Ética da Faculdade de Medicina da Universidade de Coimbra. The studies were conducted in accordance with the local legislation and institutional requirements. Written informed consent for participation in this study was provided by the participants’ legal guardians/next of kin.

## Author contributions

DS: Investigation, Writing – original draft, Writing – review & editing, Formal analysis, Methodology, Visualization. AF: Investigation, Writing – review & editing. DR: Investigation, Writing – review & editing, Validation. HP: Investigation, Validation, Visualization, Writing – review & editing. JA: Investigation, Validation, Writing – review & editing. JC: Investigation, Validation, Writing – review & editing. MS: Investigation, Validation, Writing – review & editing, Methodology, Software. MR: Investigation, Validation, Writing – review & editing. MT: Investigation, Validation, Writing – review & editing. MC-B: Investigation, Validation, Writing – review & editing, Conceptualization, Data curation, Funding acquisition, Project administration, Supervision, Writing – original draft.

## Footnotes

^1^http://www.fil.ion.ucl.ac.uk/spm
