# Supplementary material for: A neurophysiological signature of dynamic emotion recognition associated with social communication skills and cortical gamma-aminobutyric acid levels in children
Source: Front Neurosci. 2023 Dec 18;17:1295608. doi: 10.3389/fnins.2023.1295608 (PMC10757932; doi:10.3389/fnins.2023.1295608)
Supplement: Supplementary file 1 [file Data_Sheet_1.docx]

**Supplementary Information:**

**A neurophysiological signature of dynamic emotion recognition associated with social communication skills and cortical gamma-aminobutyric acid levels in children**

Daniela Sousa, Ana Ferreira, Diana Rodrigues, Helena Catarina Pereira, Joana Amaral, Joana Crisóstomo, Marco Simões, Mário Ribeiro, Marta Teixeira, Miguel Castelo-Branco

**Instruments:**

The Social Communication Questionnaire (SCQ) (Rutter et al., 2003) was developed to evaluate the history and current core symptomatology in order to screen for Autism Spectrum Disorder (ASD) from 4 to 40 years old. It is a caregiver-report screening tool that consists of 40-items of yes/no response format. It can be completed in less than 10 minutes and gives a total score with a cut-off value of 15 (i.e., the likelihood of having ASD and need for additional diagnostic evaluation) (Cronbach's alpha: .84 to .93) (Rutter et al., 2003). It also provides a total score for each one of three areas: reciprocal social interaction, communication, and repetitive/stereotyped behavior that match the Autism Diagnostic Interview-Revised (ADI-R) domains (Rutter et al., 2003). Therefore, higher scores in SCQ will correspond to higher difficulties in social communication abilities.

**Behavioral Data**

The overall accuracy mean (percentage of correct trials) for sad and happy emotional expressions was 89.24% (*SD* = 7.75). The high accuracy suggested that participants remained engaged and alert during the task. Regarding the mean of correct and error rates for happy was 89.67% (*SD* = 8.18) and .52% (*SD* = 1.17). In respect to the sad expression the mean of correct and error rates was 88.81% (*SD* = 8.16) and .29% (*SD* = .56). Other responses’ mean (i.e., before time, after time and/or no responses) was of 9.81% (*SD* = 8.28) for the happy and 10.91% (*SD* = 8.05) for the sad expressions.

**
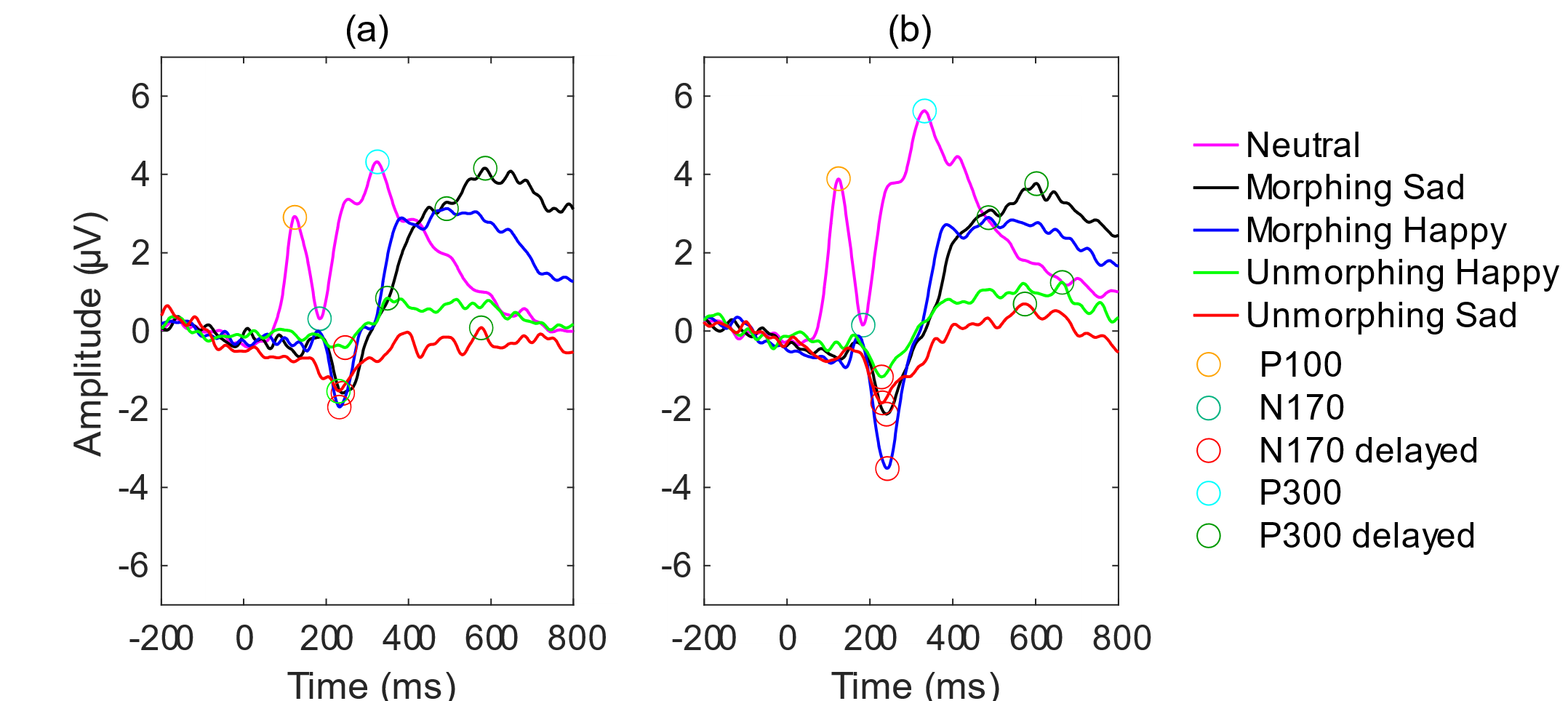
**

**Figure S1:** Grand average ERPs plots for P100, N170 and P300 components on neutral facial expression and delayed N170 and P300 at site **(a)** P3 (left hemisphere) and **(b)** P4 (right hemisphere) for emotional facial expressions.

**Figure S2**: Topographic plots for the happy facial expression at expected ERPs delayed in time N170 (on average at 250 ms) and P300 (on average at 450 ms).

**Figure S3:** Topographic plots for the sad facial expression at expected ERPs delayed in time N170 (on average at 250 ms) and P300 (on average at 450 ms).

**Figure S4**: Topographic plots for the neutral facial expression at expected ERPs P100 (100 ms), N170 (170 ms) and P300 (300 ms).

**Figure S5 :** Demonstration of the bias introduced by P100 component due to early visual features. P100-N170 peak-to-peak for neutral, happy and sad on the left (Channel P3) and right hemisphere (Channel P4).

**Figure S6:** P300 peak amplitude interaction effect between the type of stimulus (neutral and morphing), left (Channel PO7) and right (Channel PO8) hemispheres.

**Figure S7:** P300 peak amplitude interaction effect between the type of biological motion, left (Channel PO7) and right (Channel PO8) hemispheres.

**Supplementary Table 1:** Relationships between social communication skills and neurophysiological measures of facial expressions. Spearman’s rho correlations between SCQ subscales and ERP components latency (ms) and amplitude (µV) for facial expressions and hemispheres. *P-value* <.05* or *p*<.01** and FDR correction.

| ***ERP Components*** | **N170** | | **P300** |
| --- | --- | --- | --- |
|  | **Latency (ms)** | | **Amplitude (µV)** |
| ***Facial expressions***  ***(Hemisphere)*** | *Neutral*  *(Right)* | *Happy*  *(Right)* | *Sad*  *(Left)* |
| **SCQ-T** | - | rho = .44* | - |
| **SCQ-C** | - | rho = .41* | - |
| **SCQ – RSI** | rho = .39* | rho = .72** | - |
| **SCQ - R/SB** | - | - | rho = -.47* |

**Supplementary Table 2:** Associations between social communication skills and neurophysiological measures of direction of facial expression. Spearman’s rho correlations between SCQ subscales and ERP components latency (ms) and amplitude (µV) for type of biological motion and hemispheres. *P-value* <.05* or *p*<.01** and FDR correction.

| **ERP Components** | **N170** | | **P300** | | |
| --- | --- | --- | --- | --- | --- |
|  | **Latency (ms)** | | **Amplitude (µV)** | | **Latency (ms)** |
| **Direction of Facial Expression**  **(Hemisphere)** | *Neutral*  *(Left)* | *Unmorph*  *(Left)* | *Neutral*  *(Right)* | *Neutral*  *(Left)* | *Morph*  *(Right)* |
| **SCQ-T** | - | rho = .39 | - | rho = -.45* | - |
| **SCQ – RSI** | rho = .42* |  | - | - | - |
| **SCQ - R/SB** | - | rho = .47 | rho = -.39* |  | rho = .41 |

**Supplementary Table 3:** Grand averages by latency and amplitude for ERPs. The N170 and P300 ERP components for the neutral facial expression and delayed N170 and P300 for the happy and sad facial expressions by channel and for all channels (on average).

| **Channels**  **(Hemispheres)** | **Expression** | **(Delayed) N170**  **Latency (ms)**  **Amplitude (µV)** | **(Delayed) P300**  **Latency (ms)**  **Amplitude (µV)** |
| --- | --- | --- | --- |
| **F3**  **(Left)** | **Neutral** | 336 ms  -5.39 µV | 700 ms  -1.27 µV |
|  | **Happy** | 350 ms  -.49 µV | 300 ms  1.84 µV |
|  | **Sad** | 154 ms  .43 µV | 316 ms  1.38 µV |
| **F2**  **(Right)** | **Neutral** | 338 ms  -4.90 µV | 700 ms  -1.05 µV |
|  | **Happy** | 350 ms  -1.15 µV | 300 ms  .61 µV |
|  | **Sad** | 156 ms  .23 µV | 314 ms  .47 µV |
| **P3**  **(Left)** | **Neutral** | 184 ms  .31 µV | 324 ms  4.32 µV |
|  | **Happy** | 232 ms  -1.94 µV | 492 ms  3.13 µV |
|  | **Sad** | 240 ms  -1.59 µV | 586 ms  4.16 µV |
| **P4**  **(Right)** | **Neutral** | 184 ms  .15 µV | 332 ms  5.63 µV |
|  | **Happy** | 242 ms  -3.51 µV | 486 ms  2.90 µV |
|  | **Sad** | 240 ms  -2.13 µV | 602 ms  3.77 µV |
| **P7**  **(Left)** | **Neutral** | 184 ms  3.36 µV | 350 ms  4.57 µV |
|  | **Happy** | 234 ms  -2.61 µV | 494 ms  1.27 µV |
|  | **Sad** | 238 ms  -2.77 µV | 584 ms  1.53 µV |
| **P8**  **(Right)** | **Neutral** | 188 ms  2.46 µV | 362 ms  5.82 µV |
|  | **Happy** | 248 ms  -4.29 µV | 484 ms  1.36 µV |
|  | **Sad** | 234 ms  -3.62 µV | 494 ms  1.49 µV |

| **Channels**  **(Hemispheres)**  **(cont.)** | **Expression** | **(Delayed) N170**  **Latency (ms)**  **Amplitude (µV)** | **(Delayed) P300**  **Latency (ms)**  **Amplitude (µV)** |
| --- | --- | --- | --- |
| **PO3**  **(Left)** | **Neutral** | 186 ms  4.60 µV | 328 ms  8.33 µV |
|  | **Happy** | 238 ms  -3.68 µV | 486 ms  2.95 µV |
|  | **Sad** | 238 ms  -3.21 µV | 586 ms  4.78 µV |
| **PO4**  **(Right)** | **Neutral** | 182 ms  5.18 µV | 334 ms  9.89 µV |
|  | **Happy** | 242 ms  -5.11 µV | 488 ms  2.54 µV |
|  | **Sad** | 240 ms  -3.79 µV | 588 ms  3.93 µV |
| **PO7**  **(Left)** | **Neutral** | 184 ms  5.92 µV | 336 ms  8.08 µV |
|  | **Happy** | 234 ms  -4.19 µV | 496 ms  2.11 µV |
|  | **Sad** | 238 ms  -4.31 µV | 582 ms  3.20 µV |
| **PO8**  **(Right)** | **Neutral** | 182 ms  6.39 µV | 348 ms  10.40 µV |
|  | **Happy** | 242 ms  -5.87 µV | 486 ms  0.94 µV |
|  | **Sad** | 236 ms  -5.43 µV | 582 ms  2.17 µV |
| **ALL** | **Neutral**  (Mean ± Standard Error) | 245.76 (±9.82) ms  -1.51 (± .49) µV | 531.83 (±21.44) ms  1.79 (± .47) µV |
|  | **Happy**  (Mean ± Standard Error) | 267.45 (±8.88) ms  -1.67 (± .22) µV | 426.59 (±16.62) ms  1.60 (± .19) µV |
|  | **Sad**  (Mean ± Standard Error) | 250.69 (±10.37) ms  -1.17 (± .19) µV | 460.14 (±19.52) ms  1.88 (± .22) µV |
